# Perivascular epithelial cell tumor (PEComa) of the pancreas: a case report and review of previous literatures

**DOI:** 10.1186/s40792-016-0186-x

**Published:** 2016-06-15

**Authors:** Yusuke Mizuuchi, Kazuyoshi Nishihara, Akifumi Hayashi, Sadafumi Tamiya, Satoshi Toyoshima, Yoshinao Oda, Toru Nakano

**Affiliations:** Department of Surgery, Kitakyushu Municipal Medical Center, 2-1-1 Bashaku, Kokurakita-ku, Kitakyushu, Fukuoka 802-0077 Japan; Department of Pathology, Kitakyushu Municipal Medical Center, Kitakyushu, Japan; Department of Surgery, JR Kyushu Hospital, Kitakyushu, Japan,; Department of Anatomical Pathology, Graduate School of Medical Sciences, Kyushu University, Fukuoka, Japan

**Keywords:** Perivascular epithelioid cell tumor, Pancreatic neoplasm, HMB-45, Alpha-smooth muscle actin

## Abstract

Perivascular epithelial cell tumors (PEComas), firstly described by Bonetti in 1992, are a family of mesenchymal tumor composed of perivascular epithelioid cells having epithelioid or spindle morphology and exhibiting melanocytic and myogenic immunoreactivities. We herein described a 61-year-old woman who presented with epigastric pain. Preoperative imaging studies showed that 7-cm-sized mass was located in pancreatic head and body, and pancreaticoduodenectomy was performed. Histological findings showed that the tumor was composed of clear epithelioid cells with abundant glycogen granules, which grew in a nested and alveolar pattern around blood vessels. The tumor cells showed immunoreactivities for HMB-45 but did not express epithelial or endocrine markers. These histological features indicated those of PEComa. This report underlines that we should recognize PEComa as a preoperative differential diagnosis of pancreatic tumors.

## Background

Perivascular epithelial cell tumors (PEComas) are a family of mesenchymal tumors composed of perivascular epithelioid cells having epithelioid or spindle shape, clear to granular cytoplasm, centrally located, round to oval nucleus and inconspicuous nucleoli. Immunohistochemically, these neoplastic cells exhibit melanocytic marker, HMB-45, and occasionally myogenic marker, α-SMA. Angiomyolipoma, lymphangioleiomyoma, and clear cell “sugar” tumor are included in the PEComa family. PEComas arise in various organs throughout the body, especially in soft tissue, bone, abdominopelvic sites, or retroperitoneal site such as the kidney, uterus, and gastrointestinal tract, but PEComa arising in pancreas is extremely rare. We described a primary PEComa occurring in the pancreatic body of a 61-year-old woman without a history of tuberous sclerosis complexes.

## Case presentation

A 61-year-old woman was referred to our hospital with epigastralgia. Abdominal ultrasonographic examination showed 7-cm-sized mass in the head and body of the pancreas. Physical examination was unremarkable, and any symptoms were not presented except for mild epigastric tenderness. She had smoked one pack of cigarettes per day for 32 years until quitting smoking 8 years before, but not had a history of tuberous sclerosis complexes, malignancy, habitual alcohol consumption, or diabetes mellitus.

The results of blood tests were shown in Table 1. The serum levels of tumor markers showed no significant abnormal findings. As for endocrinology, serum gastrin and somatostatin levels were slightly elevated, but negative results were obtained for serum glucagon, vasoactive intestinal peptide (VIP), serotonin, and plasma immunoreactive insulin (IRI).

**Table 1:** Laboratory data

•Hematology		•Tumor marker	
WBC	4600/mm^3^	CEA	0.7 ng/ml
RBC	349 × 104/mm^3^	CA19-9	7.7 U/ml
Hb	9.9 g/dl	AFP	1.6 ng/ml
Ht	23.20 %	Elastase 1	173 ng/dl
Plt	23.2 × 104 /mm^3^	SPAN-1	11 U/ml
		DUPAN-2	<25 U/ml
•Blood chemistry			
TP	7.9 g/dl	•Endocrinology	
Alb	4.3 g/dl	Gastrin (<200)	370 pg/ml
T.Bil	0.7 mg/dl	Glucagon (40~180)	81 pg/ml
AST	27 IU/L	IRI (0~16)	7.8 μU/ml
ALT	15 IU/L	VIP (<100)	11 pg ml
LDH	265 IU/L	Serotonin (40~350)	47 ng/ml
ALP	220 IU/L	Somatostatin (1.0~12)	23 pg/ml
ɤ-GTP	12 IU/L		
Glucose	94 mg/dl	•Serology	
BUN	7.3 mg/dl	CRP	0.2 mg/dl
Cr	0.6 mg/dl		
Na	141 mEq/L		
K	4.3 mEq/L		
Cl	106 mEq/L		

**Fig. 1:**
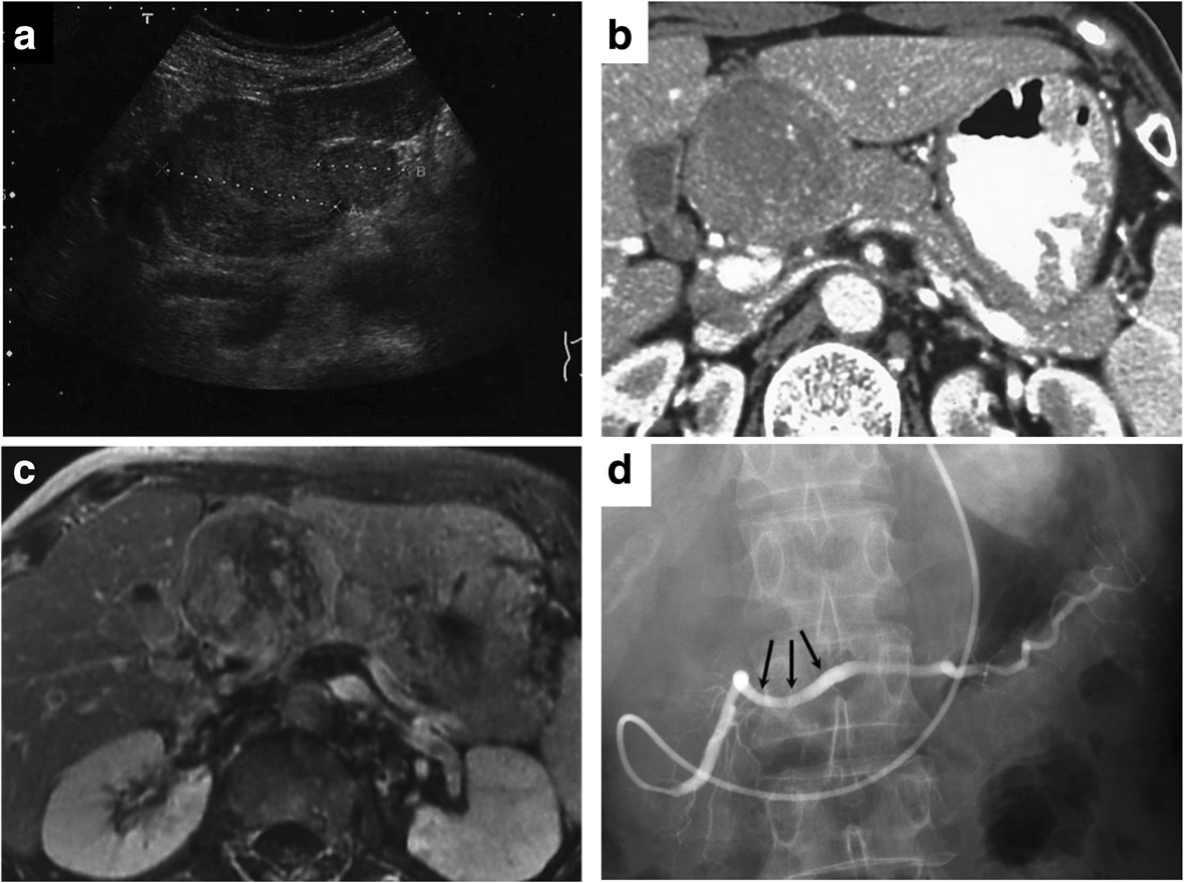
Imaging studies. **a** Ultrasonography showed that gourd-shaped hypo- and iso-echoic mass, 56 mm in size, was detected in the pancreatic head and body. **b** Enhanced computer tomography showed that solid well-circumscribed low-density mass, 7 cm in diameter, was located in the pancreatic head and body. **c** Heterogeneously enhanced mass was detected in the pancreatic body by gadolinium-enhanced MRI. **d** Endoscopic retrograde pancreatography (ERP) showed that main pancreatic duct was slightly deviated downward because of locoregional pressure effect, but direct invasion to the main pancreatic duct was not evident

Abdominal ultrasonographic examination showed that gourd-shaped hypo- and iso-echoic mass, 56 mm in size, was detected in the pancreatic head and body (Fig. 1a). Upstream main pancreatic duct was slightly dilated, 5 mm in diameter, by extrinsic compression of the mass. Neither dilatation of common bile duct nor large vessel involvement was observed.

Abdominal enhanced computed tomography (CT) scans revealed that a solid low-density mass approximately 7 cm in diameter, which was circumferentially well-demarcated, was located in the pancreatic body (Fig. 1b). The tumor was shown to be derived from the parenchyma of the pancreas. Upstream dilatation of main pancreatic duct was also noted. Neither evidences of distant metastasis nor direct invasion to adjacent organs were detected. In addition, CT scan showed that enlarged lymph nodes were not detected throughout the body including in peri-pancreatic region.

Magnetic resonance imaging (MRI) showed that the solid round mass had heterogeneous signal intensity on a T1- and T2-weighted image. In addition, gadolinium-enhanced MRI revealed that the mass was heterogeneously enhanced in the pancreatic body (Fig. 1c).

Endoscopic retrograde pancreatography (ERP) showed that main pancreatic duct was slightly deviated downward because of locoregional pressure effect, but direct invasion to the main pancreatic duct was not evident (Fig. 1d).

Neuroendocrine neoplasms, acinar cell carcinoma, and ductal adenocarcinoma variants such as clear cell carcinoma, or solid-pseudopapillary neoplasm, were suggested before operation. Since malignant tumor could not be denied according to the preoperative imaging studies, she underwent pancreaticoduodenectomy to establish a definitive diagnosis.

**Fig. 2:**
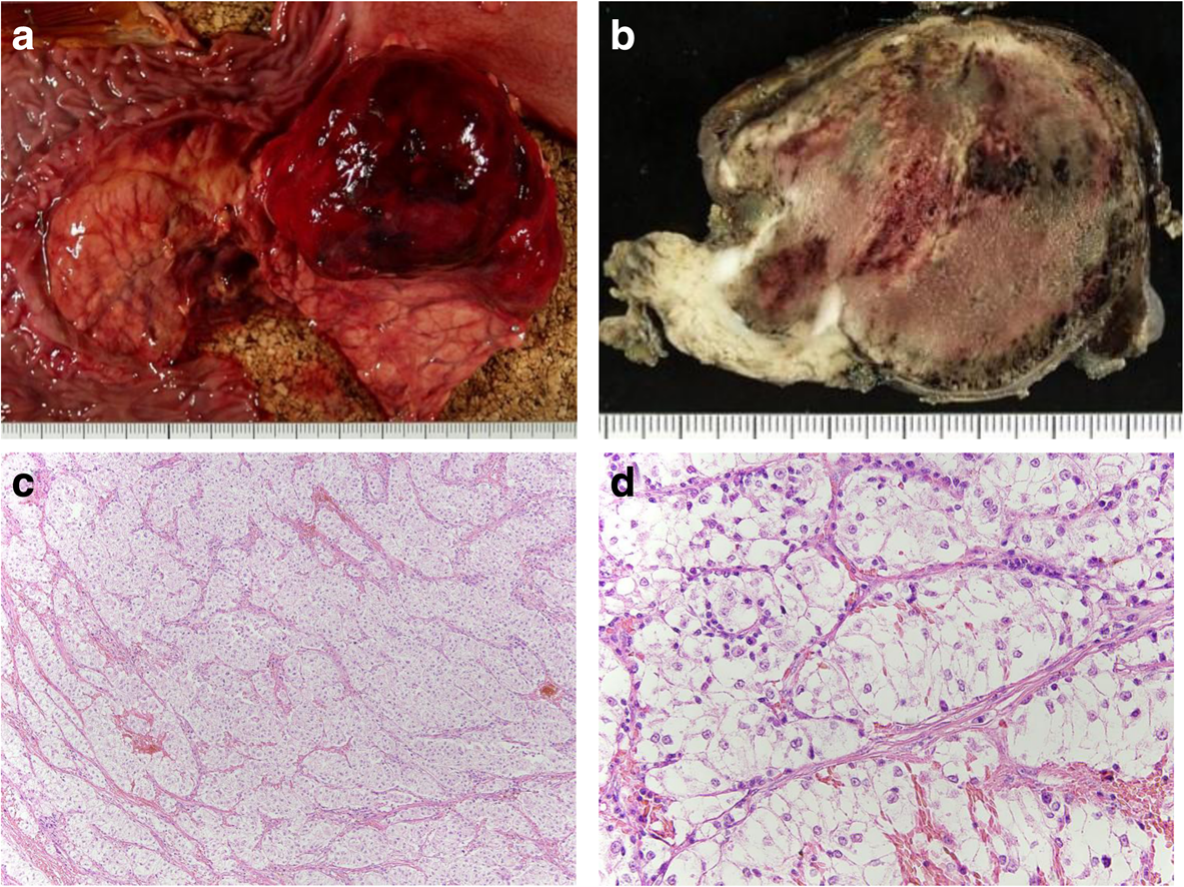
Macro- and microscopic findings of the resected specimens. **a** Well-circumscribed vaguely lobulated mass distributed mainly in the ventral side of the pancreatic head and body. **b** The cut surface of the resected specimen revealed that the brownish-colored encapsulated mass was solid associated with hemorrhage and contiguous to the pancreas. **c**, **d** Histological findings showed that the tumors were composed of perivascularly arranged epithelioid tumor cells possessing clear to focally granular eosinophilic cytoplasm, round to oval nucleus and inconspicuous nucleoli, diffusely proliferated in solid nests or alveolar pattern with sinusoidal vasculatures (hematoxylin and eosin stain C ×100, D ×400)

Macroscopically resected specimen showed a well-circumscribed vaguely lobulated mass, 6 cm in diameter, distributed mainly in the ventral side of the pancreatic head and body (Fig. 2a). The cut surface of the resected specimen revealed the brownish-colored encapsulated solid mass associated with hemorrhage at the body of the pancreas (Fig. 2b). Neither pancreatic duct change (stenosis, dilatation) nor common bile duct dilatation was evident in these specimens.

**Fig. 3:**
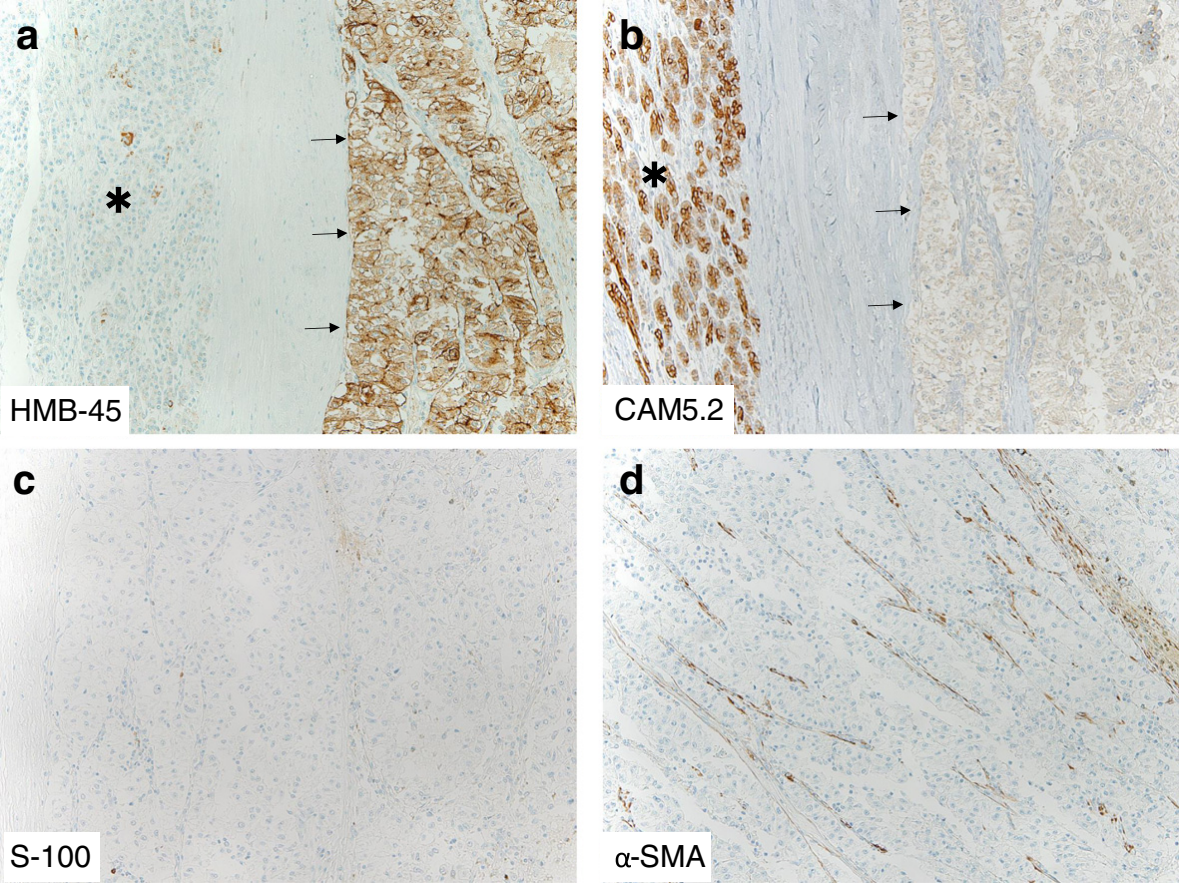
Immunohistochemical analyses. **a**, **b** The tumor cells (*arrow*) revealed cytoplasmic immunoreactivities for melanocytic marker, HMB-45, but negative for epithelial marker, CAM5.2. In normal pancreatic tissue (*asterisk*), CAM5.2 was diffusely positive, but very confined immunoreactivity was shown for HMB-45. **c**, **d** The tumor cells are negative for S-100 and α-smooth muscle actin (α-SMA). Sinusoidal vasculatures were positive for α-SMA

Microscopically, the tumors were composed of epithelioid tumor cells possessing clear to focally granular eosinophilic cytoplasm, centrally located, round to oval nucleus and inconspicuous nucleoli, diffusely proliferated in solid nests or alveolar pattern with sinusoidal vasculatures. Neither necrosis, calcification, lipomatous component, infiltrative growth pattern, mitotic figures nor vessels permeation was evident. The tumor was sharply demarcated from the pancreatic parenchyma by thick fibrous tissue (Fig. 2c, d). Results of immunohistochemical and special stain were summarized in Table 2. The tumor cells had cytoplasmic periodic acid-Schiff positive granules, which were completely digested by diastase, indicating these granules as glycogen. Immunohistochemically, these tumor cells are positive for neuron-specific enolase (NSE) and human melanoma black 45 (HMB-45), but negative for myogenic markers (α-smooth muscle actin (α-SMA) and HHF-35), epithelial markers (AE1/AE3, CAM5.2), endocrine markers (synaptophysin, chromogranin A), and acinar markers (trypsin and α-1-antitrypsin) (Fig. 3). These morphological and immunohistochemical features indicate PEComa. Ki-67 labeling index was less than 1 %.

**Table 2:** Results of immunohistochemical stain

Positive	Negative
PAS	PAS with diastase digestion
Fontana-Masson	Cytokeratins (CAM5.2, AE1/AE3)
HMB-45	Epithelial membrane antigen (EMA)
Neuron-specific enolase (NSE)	a-smooth muscle actin (a-SMA)
	Chromogranin A
	Synaptophysin
	a-1-antitrypsin
	S-100
	Vimentin
	HHF-35
	Gastrin
	Somatostatin

The postoperative course was uneventful, and she discharged 18 days after surgical operation without impaired glucose tolerance. The patient is doing well and free of recurrence 12 years after surgical resection.

### Discussion

PEComa is a term that WHO defined as “a mesenchymal tumor composed of histologically and immunohistochemically distinctive perivascular epithelioid cells (PECs)” [1]. The PECs are characterized by epithelioid or spindle-shaped appearance with a clear, eosinophilic, or granular cytoplasm, a round to oval, centrally located nucleus and an inconspicuous nucleolus. Immunohistochemical stain shows that the tumor cells are positive for HMB-45 and occasionally positive for α-SMA, S-100, and desmin, but negative for cytokeratins and endocrine markers [2]. Diffuse immunoreactivities for HMB-45 confirm that the tumor cells have melanocytic features.

By morphological and immunohistochemical similarity, PEComa family encompasses angiomyolipoma, lymphangioleiomyoma, and clear cell “sugar” tumor [3]. PEComa arise in various organs throughout the body, especially in soft tissue, bone, abdominopelvic sites, or retroperitoneal site such as the kidney, uterus, and gastrointestinal tract, but PEComa arising in pancreas is extremely rare [4]. Since Zamboni et al. firstly reported pancreatic clear cell “sugar” tumor in 1996 [5], 14 reports of this tumor family arising in pancreas were documented including this report [5–17]. Previously reported pancreatic PEComas were summarized in Table 3. The patient group consisted of 12 women and 2 men, indicating female predominance. In addition, these tumors can arise in any age of patients (age range from 17 to 74; mean age of 49.4 years) but typically found in middle-aged to elderly women. Tumors were located in pancreatic head in six patients, in pancreatic body in six patients, and in uncinated process in two patients, indicating that this tumor family could arise anywhere in the pancreas. The mean tumor size was 3.9 cm (range from 1.5 to 10 cm). Primary symptoms included abdominal pain in nine patients, diarrhea, melena, fever, and bulge in stomach in one patient, respectively. Ultrasonography for follow-up of hepatic hemangioma revealed pancreatic head mass in one patient without chief complaint [13].

**Table 3:** Summary of previous reports of pancreatic PEComa

Author	Year	Age	Sex	Tuberous sclerosis	Symptoms	Location	Operative procedure	Size (cm)	Histology	Morphological features		
Zamboni	1996	60	F	-	abdominal pain	body	DP	2	clear cell sugar tumor	epithelioid		
Heywood	2004	74	F	-	abdominal pain	uncinate process	PPPD	4.7	angiomyolipoma	epithelioid		
Ramuz	2005	31	F	-	abdominal pain	body	SPDP	1.5	sugar tumor	epithelioid		
Perigny	2008	46	F	N.A	diarrhea	body	Enucleation	1.7	PEComa	spindle > epithelioid		
Hirabayashi	2009	47	F	-	abdominal pain	head	PPPD	1.7	PEComa	spindle		
Baez	2009	60	F	-	abdominal bulge	body	DP	3.5	PEComa (sugar tumor)	epithelioid + spindle		
Zemet	2011	49	M	-	fever, cough and malaise	head	PPPD	4	PEComa	epithelioid smooth muscle cells		
Nagata	2011	52	M	-	abdominal pain	head	PD	4	PEComa	epithelioid		
Finzi	2012	62	F	-	none	head	Total excision	2.5	PEComa	epithelioid		
Al-Haddad	2013	38	F	N.A	abdominal pain	uncinate process	PD	1.8	PEComa	epithelioid + spindle		
Okuwaki	2013	43	F	-	abdominal pain	body and tail	DP	10	PEComa	spindle		
Mourra	2013	51	F	-	abdominal pain, jaund:	head	PD	6	malignant PEComa	epithelioid		
Petrides	2015	17	F	N.A	melena, anemia	head	PPPD	4.2	PEComa	epithelioid + spindle		
Our patient	2015	61	F	-	abdominal pain	body	PD	7	PEComa	epithelioid		
Glycogen granules	Necrosis	Mitosis	Ki-67 LI	Hemorrhage	α-SMA	HMB45	S-100	Desmin	Endocrine markers	Cytokeratins	Recurrence	Follow up
+	N.A	-	<1%	focally +	+	+	focally +	N.A	-	-	-	3 months
N.A	-	N.A	N.A	+	+	+	-	N.A	-	-	-	69 months
+	-	-	<1%	-	focaly +	+	focally +	-	-	-	-	9 months
+	-	1/50HPF	N.A	N.A	+	+	focally +	N.A	N.A	CK1 +	-	3 months
+	-	1/10HPF	<1%	-	+	+	+	-	-	-	-	12 months
+	-	Rare	N.A	N.A	+	+	N.A	+	N.A	-	-	7 months
+	-	-	<1%	N.A	+	+	N.A	N.A	N.A	-	-	10 months
N.A	N.A	1/50HPF	N.A	+	N.A	+	N.A	N.A	N.A	-	Liver	27 months
+	N.A	Rare	8%	N.A	+	+	focally +	N.A	-	-	-	5 months
+	N.A	N.A	N.A	N.A	+	+	-	N.A	-	-	N.A	N.A
N.A	+	N.A	<5%	+	+	+	-	N.A	N.A	-	-	7 months
+	+	2/50HPF	N.A	+	-	+	-	-	N.A	-	Liver	6 months
N.A	N.A	infrequent	N.A	N.A	+	+	-	-	-	-	-	18 months
+	-	-	<1%	+	-	+	-	-	-	-	-	12 years

*DP* distal pancreatectomy, *PPPD* pylorus preserving pancreaticoduodenectomy, *PD* pancreaticoduodenectomy

Histological diagnosis of these tumors was PEComa in ten patients, malignant PEComa in one patient, angiomyolipoma in one patient, and clear cell sugar tumor in two patients. These tumors contained varying proportions of epithelioid cell and spindle-shaped cell components [2] and associated with glycogen granules in nine patients, necrosis in two patients, and hemorrhage in six patients. In our patient, the tumor was composed mostly of epithelioid cells with clear cytoplasm, and spindle-shaped tumor cells are inconspicuous in these specimens. Glycogen granules and hemosiderin deposition are also noted, but tumor necrosis is not observed. Immunohistochemically, all the patients suffering pancreatic PEComa exhibited immunoreactivity for HMB-45 and αSMA except for two patients including our patient. S-100 immunoreactivity was observed in one patient and focally observed in four patients, and the others were negative. Desmin immunoreactivity was observed in one patient, and the others were negative as we could retrieve. Neither endocrine nor epithelial markers exhibited immunoreactivities. Myogenic marker, αSMA, exhibited immunoreactivity in spindle-shaped cells with muscular feature, and smooth muscle actin was found in 80 % of PEComa patients [2, 18]. In the present case, the tumor cells were negative for αSMA, different from other previously reported pancreatic PEComas except for one case, probably since the tumor was composed mostly of epithelioid cells in our patient [4]. Ki-67 labeling index was less than 1 % in large proportion of pancreatic PEComa patients including our patient except for one patient with less than 5 %. Prognosis of pancreatic PEComa is good, most patients remained free of relapse; however, followed-up period was only within 1 year except for the patient for 5 years. Our patient has been no sign of recurrence for 12 years. Thus, our case is the first report of long-term follow-up of PEComa of the pancreas over 10 years.

Differential diagnoses of pancreatic PEComa were included epithelial tumors such as primary clear cell carcinoma, metastatic renal cell carcinoma, endocrine tumor with clear cell change, solid pseudopapillary neoplasm, and acinar cell carcinoma. It is relatively easy to distinguish this entity from the other possible diseases in surgical resected specimen by immunohistochemical study for epithelial markers, neuroendocrine markers, acinar markers, myogenic markers, and HMB-45. Recently, clear cell sarcoma arising in pancreas was reported by Huang et al. [19]. This rare malignant soft tissue neoplasm affects young adult, has a poor prognosis, and expresses melanocytic marker, HMB-45. We could rule out this tumor entity because S-100 protein immunoreactivity was low in the tumor of our patient.

Tuberous sclerosis is a systemic genetic disease that caused seizures, intellectual disability, and benign tumor development in multiple organs including the brain and skin [2, 20, 21]. Recent study mentioned that mTOR inhibitor was effective in PEComa associated with tuberous sclerosis because of mTOR pathway upregulation [22]. Although many lesions of PEComa family observed in tuberous sclerosis complex patients, there are no pancreatic PEComa patients having a history of tuberous sclerosis complexes to the extent that we could retrieve, possibly suggesting that pancreatic PEComa has little association with tuberous sclerosis complexes.

Folpe et al. reported in 2005 that the presence of tumor recurrence or metastasis was predicted by worrisome features in PEComa arising from soft tissue and gynecologic origins [2]. Worrisome features included large size (>5 cm), infiltrative growth, high nuclear grade, hypercellularity, necrosis, and high mitotic figures (>1/50 HPF). Previous reports in pancreatic PEComa revealed liver metastasis in two patients. When we adapted these worrisome features to pancreatic PEComa and retrieved these features as we can from the literatures about these two recurrent tumors, we found necrosis in one patient, and mitotic figures are sparsely seen (2/50HPF and 1/50HPF, respectively), but the other features are lacking in these tumors. In our patient, the tumor is 6 cm in size and mitoses are seen (1/50HPF), thus applicable to two worrisome features of PEComa arising from the soft tissue and gynecologic organs, but our patient is free of recurrence 12 years after surgery.

As pancreatic PEComa is difficult to diagnose preoperatively, extensive resection with lymph nodes dissection such as pancreaticoduodenectomy and distal pancreatectomy was performed in 12 patients including our patients, whereas two patients underwent local excision. Lymph node relapse was not seen in the patients of pancreatic PEComa, suggesting that lymph node dissection might be omitted in pancreatic PEComa.

In our patient, serum levels of gastrin and somatostatin were slightly elevated in contrast to the other reports of PEComa, but the cause of elevating these hormones is not determined in other blood tests and imaging studies. Furthermore, immunohistochemical staining revealed the tumor cells were negative for gastrin or somatostatin.

Since clinical experience of pancreatic PEComa is very limited, the accumulation of this disease’s reports is required to define the worrisome features as an indicator of recurrence and metastasis of pancreatic PEComa, and since the tumor can recur, we recommend complete resection and careful follow-up.

## Conclusions

We reported the patient with PEComa arising in pancreatic body with characteristic immunohistological features. We suggest that clinicians should be aware of pancreatic PEComa in diagnosing a patient with pancreatic mass, if there are findings suspicious for neoplasm with clear cell features including clear cell carcinoma, acinar cell carcinoma, or solid pseudopapillary neoplasm in preoperative imaging studies or EUS-guided biopsy samples.

## Consent

Written informed consent was obtained from the patient for the publication of this Case Report and any accompanying images. A copy of the written consent is available for review by the Editor-in-Chief of this journal.

## Abbreviations

CT, computed tomography; ERP, endoscopic retrograde pancreatography; HMB-45, human melanoma black 45; IRI, immunoreactive insulin; MRI, magnetic resonance imaging; NSE, neuron-specific enolase; PEComas, perivascular epithelial cell tumors; PECs, perivascular epithelioid cells; VIP, vasoactive intestinal peptide; α-SMA, α-smooth muscle actin
